# Improving gender‐affirming care in genetic counseling: Using educational tools that amplify transgender and/or gender non‐binary community voices

**DOI:** 10.1002/jgc4.1581

**Published:** 2022-04-23

**Authors:** Nicole Huser, Bailey B. Hulswit, Diane R. Koeller, Beverly M. Yashar

**Affiliations:** ^1^ Department of Human Genetics University of Michigan Ann Arbor Michigan USA; ^2^ Holden Comprehensive Cancer Center University of Iowa Iowa City Iowa USA; ^3^ University of Michigan Rogel Cancer Center, 1500 E. Medical Center Dr Ann Arbor MI USA; ^4^ Division of Cancer Genetics and Prevention Dana‐Farber Cancer Institute Boston MA USA

**Keywords:** community, cultural competence, education, gender‐affirming, non‐binary, transgender

## Abstract

Transgender and/or gender non‐binary (TGNB) individuals face significant health care disparities, including deficiencies in provider knowledge. To address this knowledge gap for genetic counselors, we developed, implemented, and analyzed an educational intervention on gender‐affirming genetic counseling (GC) and care for TGNB patients. In partnership with the TGNB community, we designed a 5‐module (length = 146 min ± 94 min) genetic counseling‐targeted online learning program focused on gender‐affirming care (*Amplify*). Content included elements of gender‐affirming care, core components of gender‐inclusive GC sessions, and cancer risk assessment/management. Video testimonials featuring TGNB individuals complemented learning within each module. Educational outcomes measured included comfort working with TGNB patients (*n* = 2 multiple choice questions (MCQs)), impact of education on knowledge (*n* = 25 MCQs), and clinical self‐efficacy based on the Accreditation Council for Genetic Counseling competencies (*n* = 35 skills). Participants (*n* = 40), recruited through state and national GC organizations, completed all modules, and pre‐ and post‐education/self‐efficacy assessments. Pre‐*Amplify*, 65% (*n* = 26/40) of participants endorsed feeling ‘somewhat comfortable’ working with TGNB patients. The average knowledge score was 77.6% (*SD* = 11.2%) with the lowest scores related to the gender affirmation process. After *Amplify*, overall knowledge improvement was statistically significant with an average 16.9% (*p* < 0.001) increase in score. Pre‐*Amplify*, the average self‐efficacy score was 78.4% (*SD* = 15.8%) with lowest scores seen in statements surrounding information gathering of family and medical histories. Post‐*Amplify*, overall self‐efficacy improvement was statistically significant with an average 13.8% (*p* < 0.001) increase in score. Linear regression did not identify an impact of practice specialty on participants’ knowledge gains or self‐efficacy. This study shows online modules are an effective form of gender‐affirming care education for GCs. This intervention can positively improve the care practicing genetic counselors provide to patients and inform future decision‐making about the development of gender‐affirming care education for genetic counselors.

**What is known about this topic**

Genetic counselors lack comfort in some aspects of gender‐affirming communication practices and assessing cancer risks of TGNB patients. Genetic counselors desire more education on gender‐affirming genetic counseling and care for TGNB patients.

**What this paper adds to the topic**

This paper describes the development, implementation, and analysis of an online learning program that is focused on providing gender‐affirming care by genetic counselors. It highlights the value of TGNB community member engagement in genetic counselor education and the positive impact TGNB‐targeted education could have on genetic counselors in varied clinical settings.

## 1 INTRODUCTION

Transgender and/or gender non‐binary (TGNB) individuals face significant health care disparities, and in turn, experience poorer health outcomes. This has been observed in refusal of care, being asked invasive questions unrelated to the purpose of the health care visit, and/or deficiencies in health care provider knowledge on appropriate care needs (James et al., 2016). In a 2011 survey of over 6,000 transgender individuals in the United States, 50% of participants reported having to educate their health care providers about transgender health care needs (Grant et al., 2011). This knowledge gap leads to avoidance of the health care system by TGNB patients. Individuals who had to teach health care providers about transgender people were found to be four times more likely to delay necessary health care services (Jaffee et al., 2016). Additionally, providers may fail to recognize the importance of eliciting gender identity in a health care encounter. In the 2015 U.S. Transgender Survey, 31% of participants reported none of their health care providers knew they were transgender (James et al., 2016).

There have been limited studies exploring genetic counseling practices with TGNB patient populations. The published work describes genetic counselors’ (GCs) general experiences working with TGNB patients (Sacca et al., 2019; Zayhowski et al., 2019) as well as GC comfort and knowledge in estimating appropriate cancer and disease risks (Sutherland et al., 2020; Vaupel‐Klein & Walsh, 2020). Multiple studies have shown a high percentage of GCs (91% (*n* = 198), 98.6% (*n* = 437) respectively), desire more education on TGNB health care and implications on cancer risks (Berro et al., 2019; Sheehan et al., 2020). There is also limited professional consensus around pedigree nomenclature for TGNB individuals (Barnes et al., 2019; Sheehan et al., 2020; Tuite et al., 2020). Finally, while the impact of obtaining TGNB perspective and guidance has been highlighted (Barnes et al., 2019), TGNB community advisory boards have not been utilized by the genetic counseling community. A major theme from a study eliciting transgender and gender‐diverse individuals’ perspectives on trans‐associated genetic research was TGNB community involvement at all levels of the research process and utilization of diverse community advisory boards (Rajkovic et al., 2022).

Looking to TGNB care education practices in other health care professions, various methods have been employed but there is no consensus on the most effective method of providing education on transgender health topics (Dubin et al., 2018). All analyzed approaches (e.g., mandatory module, curricular content, standardized patients, clinical observation, optional lunch elective) in this comprehensive review were associated with improving attitudes, knowledge, and/or skills necessary to reach clinical competency; however, the most common approach, a one‐time lecture, was only associated with short‐term improvement outcomes. Single interventions often focus on attitudes and awareness with limited education on clinical skills and improving self‐efficacy (Donaldson & Vacha‐Haase, 2016; Dubin et al., 2018; Israel et al., 2014). While some of the content that has been used to educate other health care providers on gender‐affirming care can be applied to GCs, i.e., inclusive language and TGNB population disparities, GCs have unique education needs related to inheritance, generating pedigrees, and completing genetic risk assessments for patients and families in a gender‐inclusive manner.

This study describes the development, implementation, and analysis of an educational intervention (*Amplify*) for genetic counselors on gender‐affirming TGNB care that targets both knowledge gaps and clinical competence. Changes in knowledge of a wide range of TGNB health care topics were assessed, along with comfort and self‐efficacy in providing genetic counseling to TGNB patients independent of clinical specialty. Our analysis of GCs overall competence and confidence demonstrates *Amplify's* effectiveness and illuminates how this education can transfer into improved patient care and inform future approaches to gender‐affirming care education for GCs and trainees.

## 2 METHODS

This study was granted exemption status from the University of Michigan Institutional Review Board (HUM00186581).

### 2.1 Community advisory board development

A community advisory board (CAB) was developed that brought together a diverse group of individuals from the TGNB community with the purpose of ensuring that consistent community member perspective and feedback was available to help guide decision‐making on the entire project. The CAB was critical due to the fact gender‐inclusive terminology and health care needs of the TGNB community are rapidly evolving. Four individuals were recruited through local (ex: University of Michigan Spectrum Center) and national (ex: National Organization of Gay and Lesbian Scientists and Technical Professionals) TGNB social platforms to ensure variations in age, gender identity, racial and ethnic background, and geographic location. Monthly meetings with the project team were used to review developed content and discuss emerging questions related to gender‐affirming care and genetic counseling. CAB members were compensated for time contributed to this project.

### 2.2 Development online educational platform

Content for the online educational modules (*Amplify*) was developed following a thorough review of the literature (Deutsch, 2016; Hembree et al., 2017; LGBTQIA+Health Education Center, 2020; World Professional Association for Transgender Health, 2012) and synthesized expert perspectives including endocrinology, social work, and genetic counseling (local and national). Diverse TGNB community members shared their expertise and lived experience. Experts were utilized in informational interviews and content review. *Amplify* was designed to provide learners with a foundation of gender‐affirming communication skills, insights that can be used to examine their clinical practice for gender inclusivity, as well as broaden their overall awareness of the TGNB community. Bloom's Taxonomy was utilized as a framework in constructing learning objectives for each module (Forehand, 2005). Modules were created to enable learners to build knowledge consecutively through the course: beginning with a detailed review of terminology and finishing with a critical analysis of the components of a genetic counseling session. A summary of current knowledge regarding cancer risk assessment of TGNB patients was also included due to the expressed need in prior literature (Berro et al., 2019; Sacca et al., 2019; Sutherland et al., 2020; Zayhowski et al., 2019). The module titled ‘Pulling it All Together ‐ The Genetic Counseling Session’ was intended to aid in building clinician self‐efficacy. Additionally, practice quiz questions and interactive learning activities were included which had been reviewed by the CAB. A detailed breakdown of the content in each module can be found in Table 1.

**TABLE 1:** Online learning module and Slack channel content

Time estimate	Module title	Content description of modules
25 min	Terminology, Population Disparities, and Communication	A comprehensive glossary, breakdown of TGNB population disparities, as well as gender‐affirming communication considerations for the genetic counselor
20 min	Clinical Environment	An examination of the clinical environment from the patient perspective, from start to finish, including things such as in‐take forms, the physical environment, and documentation
25 min	Potential Aspects of the Gender Affirmation Process	A summary of potential psychological, social, hormonal, surgical, and legal affirmations individuals make undergo in their gender affirmation process
40 min	Cancer Risk Assessment	An organ‐based perspective summarizing what is known up to this point in the literature on cancer risks of TGNB individuals and how to consider possible hormonal or surgical affirmations into risk estimates and management
30 min	Pulling it All Together‐ The Genetic Counseling Session	Applying everything in Modules 1–4 to core components of the genetic counseling session such as family and medical history gathering, education, psychosocial considerations, and patient resources

	Slack thread title	Purpose of discussion thread
	General Discussion	Propose any topic to the group around gender‐affirming care in genetic counseling as well as quiz questions and polls to engage the group by the project director
	Overcoming Stereotypes and Biases	Continue the conversation of how we can overcome implicit biases and stereotypes of the TGNB community
	Clinical Environment	Bring forward strategies to make the clinical environment gender‐inclusive
	Cancer Case Example	Discuss thoughts to the questions posed by the case example in Module 4 as well as any examples a participant seeks broad group feedback on.
	Resources	Find and share resources of organizations that advocate for members of the TGNB community

Abbreviation: TGNB, transgender and/or non‐binary.

To broaden participant awareness of the TGNB community, amplify TGNB voices, and highlight module content, each module includes video testimonials (length 20 s‐3 min) from four diverse TGNB community members, two were CAB members. In total, *Amplify* includes 28 testimonials.

All content and videos were collated into online modules in the program Articulate 360‐Rise 360, exported for SCORM 2004, and imported to a Learning Management System (LMS) Canvas page supported by the University of Michigan. This allowed the project team to track participant access to the program, total training time, and number of page views per participant. The entire program was designed to be self‐paced and take 60–90 min to complete.

Participants were provided the option of joining a private online discussion forum hosted on Slack, a business communication platform. The purpose of this group was to provide a space for open reflection around the content in *Amplify*, discussion of gender inclusivity in the genetic counseling profession, development of shared resources, and interaction with the CAB by posing questions. It was divided into five separate threads (Table 1) to focus conversations and maximize utility of the platform for participants. The study team monitored engagement in the Slack community by counting the number of participant posts on each thread and noting commonly discussed topics. In order to foster relationships and support individual accountability, participants were identifiable.

### 2.3 Participants and procedures

Participants were recruited through the National Society of Genetic Counselors (NSGC) Student Research Survey Program, American Board of Genetic Counseling (ABGC) Student Research Request, and Michigan Association of Genetic Counselors (MAGC) member listserv. Qualifying participants were board‐eligible or certified GCs. A small number of genetic counseling students were recruited through the MAGC listserv. Recruitment occurred between November 2020 and January 2021 during the COVID‐19 pandemic.

Study eligibility (practicing GCs or genetic counseling students) was determined by a screening survey that collected demographic information, prior TGNB education, and comfort caring for and assessing TGNB patient cancer risks. Eligible participants were required to complete the knowledge and genetic counseling self‐efficacy (GCSE) assessment prior to and after completion of *Amplify*. They had 9 weeks to complete the developed educational modules.

### 2.4 Instrumentation

#### 2.4.1 Demographics and comfort assessment

We developed a screening survey (*n* = 25 total multiple choice questions (MCQs)) that evaluated GC status, years of practice, specialty, patient load, and exposure to the TGNB community, both personally and professionally. Questions also evaluated age, gender identity, sexual orientation, race, region, graduate program attended, and year of graduation to determine if the sample was representative of the genetic counseling profession. Participants identified prior TGNB education and self‐reported comfort caring for TGNB patients and assessing TGNB patient cancer risks (*n* = 2 MCQs). Of note, the Merriam‐Webster Dictionary defines comfortable as ‘free from stress or tension’ or ‘free from vexation or doubt’ (Merriam‐Webster, n.d.).

#### 2.4.2 Knowledge and self‐efficacy assessment

To assess knowledge, the project team developed 25 MCQs in the knowledge and GCSE assessment which were based on *Amplify* module content (five MCQs/module) with a maximal total knowledge score of 25 (see Table 1). Total and module‐specific knowledge scores were calculated for each participant based on the number of correct MCQs answered. To assess GCSE in working with TGNB patients, the authors adapted the validated GCSE scale to identify the TGNB patient population in each assessed competency (Caldwell et al., 2018; Keller et al., 2019). Utilizing the adapted scale, participants rated how certain they were on a scale of 0–100 they could currently, independently perform 35 different competencies in a genetic counseling session with a patient who identified as TGNB. These competencies were divided into six categories for analysis.

The 25 MCQs related to knowledge and the 35 in the GCSE were combined to create the 60 MCQ online assessment that was completed before and after *Amplify*. Three additional open‐ended questions were asked at the end of the post‐education assessment collecting qualitative feedback on the online module user experience as well as possible interest in an interactive, virtual event alongside other GCs and members of the TGNB community. The pre‐ and post‐test survey responses for each participant were linked using participant email, then immediately deidentified with a randomized participant ID number.

### 2.5 Data analysis

Descriptive statistics were used to summarize demographic information from the screening survey along with data obtained regarding prior TGNB education and comfort working with TGNB patients. Categorical variables were reported with frequencies (percentages). Specialty of practice was recoded for each participant as cancer (1) or non‐cancer (0). Personal exposure to the TGNB community was recoded for each participant as ‘yes’ (1) or ‘maybe/no’ (0). Knowledge scores were summed, both overall out of 25, and individually within each of the module content groups out of five, for each participant. These scores were converted to percentages and a mean and standard deviation were calculated for both the pre‐ and post‐education assessments. GCSE scores were averaged overall out of the 35 competencies to an overall score of GCSE working with TGNB patients. Mean and standard deviation were calculated of this overall score as well as within each of the six competency categories for both the pre‐ and post‐education assessments.

Linear regression was performed to determine if personal exposure to the TGNB community was a significant predictor of knowledge before the *Amplify* educational intervention and GCSE. Linear regression was also completed to determine if specialty of practice was a significant predictor of overall change in knowledge or GCSE from the *Amplify* modules. A nominal *p* value threshold (*p* < 0.05) was applied for significance. Paired *t*‐tests were performed to compare the mean differences in knowledge and GCSE scores between pre‐education assessment and post‐education assessment. A Bonferroni correction was used to control for a Type 1 error rate across the 13 paired *t*‐tests and an adjusted *p* < 0.0038 was applied for significance. The analysis was performed using SPSS software with assistance from the University of Michigan Consulting for Statistics, Computing and Analytics Research (CSCAR).

## 3 RESULTS

A total of 95 GCs and three genetic counseling students responded to the initial survey for this study. Sixteen were not interested in moving forward with the study or did not complete the pre‐education knowledge/GCSE assessment. Eighty‐two participants completed the pre‐education assessment, and of those, 40 completed the post‐education assessment. The presented pre‐ and post‐test analyses describe the 40 participants who completed both assessments.

### 3.1 Demographics

Most participants were certified GCs (95.0%) and cisgender women (95.0%) (Table 2). Of the 40 GCs, most were heterosexual (82.5%) and white (90.0%) between the ages of 25 and 34 (60.0%). A majority had been practicing genetic counseling for under five years (72.5%) and 35% were currently practicing in the cancer specialty. Prior experience working with a patient who is TGNB in the last year varied widely (0–15 patients) and 55% said ‘yes’ when asked if a patient had ever self‐disclosed to them that they identified as TGNB. Almost two‐thirds (62.5%) of participants personally knew someone in the TGNB community.

**TABLE 2:** Demographics

Variable	*n*	%
Genetic counselor status		
Certified genetic counselor	38	95.0
Board‐eligible genetic counselor	1	2.5
Student	1	2.5
Gender identity		
Cisgender woman	38	95.0
Cisgender man	1	2.5
Gender non‐conforming woman	1	2.5
Sexual orientation		
Heterosexual	33	82.5
Homosexual	4	5.0
Bisexual	2	10.0
Queer	1	2.5
Race		
White	36	90.0
Asian	1	2.5
Asian Indian	1	2.5
Black or African American	1	2.5
Middle Eastern and White	1	2.5
Age		
18–24	4	10.0
25–34	24	60.0
35–44	9	22.5
45–54	2	5.0
55–64	1	2.5
Total years of experience practicing genetic counseling		
0–5	29	72.5
6–10	4	10.0
11–15	2	5.0
16–20	2	5.0
21–25	2	5.0
Current student	1	2.5
Region^a^		
Region I	2	5.0
Region II	8	20.0
Region III	2	5.0
Region IV	23	57.5
Region V	2	5.0
Region VI	3	7.5
Specialty		
Cancer	14	35.0
Non‐cancer	26	65.0
TGNB patients seen in practice in the past year		
0	12	30.0
1–3	16	40.0
4–6	9	22.5
7–9	0	0.0
10–12	1	2.5
13–15	2	5.0
A patient has disclosed to you they identify as TGNB during a genetic counseling session		
Yes	22	55.0
Maybe	0	0.0
No	18	45.0
Education on TGNB care in graduate training		
Yes	15	37.5
Maybe	7	17.5
No	18	45.0
TGNB involvement in education in graduate training^b^		
Yes	3	20.0
Maybe	7	46.7
No	5	33.3
Personally knows someone in the TGNB community		
Yes	25	62.5
Maybe	2	5.0
No	13	32.5

Note

TGNB, transgender and/or non‐binary.

^a^ Region I: CT, MA, ME, NH, RI, VT, CN Maritime Provinces, Region II: DC, DE, MD, NJ, NY, PA, VA, WV, PR, VI, Quebec, Region III: AL, FL, GA, KY, LA, MS, NC, SC, TN, Region IV: AR, IA, IL, IN, KS, MI, MN, MO, ND, NE, OH, OK, *SD*, WI, Ontario, Region V: AZ, CO, MT, NM, TX, UT, WY, Alberta, Manitoba, Saskatchewan, Region VI: AK, CA, HI, ID, NV, OR, WA, British Columbia

^b^ Only participants who answered ‘yes’ to TGNB education in graduate training were asked about TGNB involvement in development and administration of said education.

### 3.2 Prior education on TGNB care

The majority of participants (45%) stated they had no specific TGNB training in their graduate education, and seven individuals were unsure (20%). Those who did have this training (35%) were asked if individuals in the TGNB community were involved in the development or administration of said education and 20% responded ‘yes’ (Table 2).

### 3.3 Comfort of GCs

When asked how comfortable participants felt working with a patient who identified as TGNB, a majority endorsed feeling ‘somewhat comfortable’ (65.0%) and 17.5% felt ‘extremely comfortable’. The remaining participants stated they were ‘neither comfortable nor uncomfortable’ or ‘somewhat uncomfortable’ (17.5%). Additionally, when asked how comfortable participants were assessing the cancer risk of a patient that identified as TGNB, a majority endorsed feeling ‘somewhat comfortable’ (47.5%) and 7.5% felt ‘extremely comfortable’. The remaining participants (45%) stated they were ‘neither comfortable nor uncomfortable’ or ‘somewhat uncomfortable’ assessing cancer risk for these patients.

### 3.4 *Amplify* user experience

Participants’ learning experiences within *Amplify* were quite variable. The mean time participants spent in *Amplify* was 146 min (*min* = 37 min, *max* = 509 min, *SD* = 94 min). The mean number of page views per participant was nine (*min* = 2 views, *max* = 40 views, *SD* = 7 views). Participant feedback on their experiences working through *Amplify* highlighted the value of the content and the presentation:

I wish I had more of this instruction when I was in genetic counseling school and that all our institutions were aware of the exclusivity that many health care spaces communicate.

I thought the videos were an extremely valuable part of the program, but I think having them in small clips added much more value than in one longer video…The interactive nature of this program kept me engaged the whole time and really made me focus on the content… I would love for this course to be more widely available.

Voices amplified through this project will stay with me as I care for future patients.

### 3.5 Slack community user experience

The Slack threads with the most participant posts were the ‘Overcoming Stereotypes and Biases’ thread, followed by ‘Clinical Environment’. Common themes brought up in these threads included how personal connections with the TGNB community interplay with biases in the clinic, reactions to implementing gender‐affirming communication strategies into counseling practice, as well as efforts to make gender‐inclusive changes to the genetic counseling clinic. The thread with the least engagement was ‘Cancer Case Example’ with no participant posts or engagement. Participants appeared to be most likely to engage within Slack when a direct post was not required, for example, responding to an anonymous quiz question or poll put out to the entire group on the ‘General Discussion’ thread.

### 3.6 Knowledge of gender‐affirming TGNB care

Gender‐affirming TGNB care knowledge was assessed prior to participants gaining access to *Amplify*. Results from statistical analyses relating to knowledge can be found in Table 3. The average pre‐education knowledge score of participants was 77.6% (*SD* = 11.2%). Participants had the lowest average score of 61.0% (*SD* = 23.5%) on the module content of ‘Potential Aspects of the Gender Affirmation Process’. The content participants had the strongest pre‐*Amplify* knowledge in was ‘Terminology, Population Disparities, and Communication’ at 92.5% (*SD* = 11.7%). Personal exposure to the TGNB community was not a significant predictor of pre‐education knowledge (*p* = 0.131). After *Amplify*, the average post‐education knowledge score of participants was 94.5% (*SD* = 4.5%). There was a statistically significant average improvement of 16.9% (*SD* = 11.6%; *p* < 0.001). Significant improvements were also observed in each module of gender‐affirming TGNB care knowledge. The module with the greatest change in score between the pre‐ and post‐education assessments was ‘Potential Aspects of the Gender Affirmation Process’ with an average increase in score of 32.5% (*SD* = 25.5%; *p* < 0.001).

**TABLE 3:** Results of knowledge assessment

Module Content	Paired differences						
			95% Confidence interval of the difference				
	Mean	SD	Lower	Upper	t	*df*	*p*‐value*
Potential Aspects of Gender Affirmation	32.5	25.5	24.3	40.7	8.1	39	<0.001
Cancer Risk Assessment	19.5	20.5	12.9	26.1	6.0	39	<0.001
Genetic Counseling Session	15.0	19.6	8.7	21.3	4.8	39	<0.001
Clinical Environment	11.0	16.3	5.8	16.2	4.3	39	<0.001
Terminology, Population Disparities, Communication	6.5	13.1	2.3	10.7	3.1	39	0.003
Overall Knowledge	16.9	11.6	13.2	20.6	9.2	39	<0.001

* An adjusted *p* < 0.0038 was used to assess for significance in the performed paired *t*‐tests.

### 3.7 Genetic counseling self‐efficacy with TGNB patients

GCSE with TGNB patients was assessed prior to participants gaining access to *Amplify*. Results from statistical analyses relating to GCSE can be found in Table 4. The average pre‐education GCSE with TGNB patients was 78.4% (*SD* = 15.8%). Personal exposure was not a significant predictor of pre‐*Amplify* GCSE (*p* = 0.640). Participants had the lowest average GCSE of 72.5% (*SD* = 20.1%) in the competency of ‘Information Gathering’. The competency participants had the highest GCSE in was ‘Genetic Counseling Process’ at 87.1% (*SD* = 15.0%). After *Amplify*, the average post‐education GCSE with TGNB patients was 92.2% (*SD* = 8.0%). There was a significant average improvement of 13.8% (*SD* = 11.1%; *p* < 0.001). Significant improvements were also observed in each GCSE competency. The module with the greatest change in GCSE between the pre‐ and post‐education assessments was ‘Information Gathering’ with an average increase of 19.5% (*SD* = 16.1%; *p* < 0.001).

**TABLE 4:** Results of genetic counseling self‐efficacy assessment

Competency category	Paired differences						
			95% Confidence interval of the difference				
	Mean	SD	Lower	Upper	t	*df*	*p*‐value*
Information Gathering	19.5	16.1	14.3	24.6	7.7	39	<0.001
Case Management	17.7	13.6	13.3	22.0	8.2	39	<0.001
Psychosocial Counseling	15.2	13.6	10.8	19.6	7.1	39	<0.001
Genetic Testing	13.8	13.0	9.7	18.0	6.7	39	<0.001
Communication	11.1	11.9	7.3	14.9	5.9	39	<0.001
Genetic Counseling Process	7.7	10.4	4.3	11.0	4.7	39	<0.001
Overall GCSE with TGNB Patients	13.8	11.1	10.3	17.4	7.8	39	<0.001

* An adjusted *p* < 0.0038 was used to assess for significance in the performed paired *t*‐tests. TGNB: transgender and/or non‐binary. GCSE: genetic counseling self‐efficacy.

### 3.8 Effectiveness across specialties

We wanted to assess if the developed education could be effective for GCs practicing in any specialty. When analyzing if specialty of practice (cancer or non‐cancer) was a significant predictor of change in overall knowledge score post‐education compared to pre‐education, no statistical significance was observed (*p* = 0.562). This was also the case when assessing if specialty was a significant predictor of change in overall GCSE working with TGNB patient's post‐education compared to pre‐education (*p* = 0.750).

## 4 DISCUSSION

This is the first study of its kind to develop an educational intervention for GCs around gender‐affirming TGNB care and analyze education effectiveness in the domains of competence and confidence. GCs had the lowest knowledge pre‐*Amplify* around potential components of the gender‐affirmation process and subsequently, the greatest improvement in knowledge of that subject. Overall GCSE and knowledge were significantly improved regardless of practice specialty of the GC. Our data showed GCs had a strong background in TGNB‐related terminology and disparities faced by the TGNB community, with greater personal exposure to the community when compared to professional settings. Participants cited the inclusion of TGNB perspectives in the online modules as a valuable component which highlights the importance of involving TGNB community members in the development of educational content.

A striking finding from the pre‐*Amplify* data was that GCs had a low initial knowledge and awareness around potential components of the gender‐affirmation process (61.0%) compared to terminology, population disparities, and communication (92.5%). This gap in knowledge highlights the need for this subject matter to be included in future educational material developed for GCs. Strengthening this knowledge base will aid in refining interpretations of altered disease risk profiles for TGNB patients following potential hormonal and surgical gender affirmations. This category of gender‐affirming TGNB care knowledge had the highest change in score with an average improvement of +32.5% post‐*Amplify*. This improvement may have been due to the TGNB community member voices providing detailed accounts of their own personal journeys and expanding awareness of the extreme diversity of individuals in this population. Pairing relevant content for GCs in tandem with voices of the TGNB community highlights the significance of the education being provided.

Our results demonstrate that online learning modules are an effective means of improving GC knowledge and GCSE no matter their specialty of practice. Knowledge was significantly improved on average by +16.9% and GCSE by +13.8%. *Amplify* had content specifically focused on the cancer specialty as prior literature had identified a need for more TGNB care education in this sub‐specialty (Berro et al., 2019; Sheehan et al., 2020). However, GCs from all major specialties were interested in learning about gender‐affirming TGNB care, in fact, 65% of the study participants were currently practicing in a specialty other than cancer. The fact that there were no statically significant differences in the degree to which knowledge was improved for participants based on their clinical specialty demonstrates the utility of the *Amplify* education platform for all providers and highlights the need for this type of education within the genetic counseling profession as a whole. Research outside of the cancer specialty has shown a need for more gender‐affirming TGNB care education in the preconception and prenatal genetic counseling settings. A qualitative study found gendered language is common practice in these specialties with routinely used phrases including: *advanced maternal age, mom/mother,* and *dad/father,* which do not always accurately describe the patient or family system in a genetic counseling session (Ruderman et al., 2021). Future educational interventions in this domain could be impactful in cancer and prenatal genetics, but also in the well‐established genetic counseling specialties of pediatric, cardiology, and emerging specialties of neurology and nephrology.

Gender‐affirming care education, like *Amplify,* can provide GCs with a more well‐rounded perspective on the TGNB community. Within the assessment of knowledge pre‐*Amplify* around gender‐affirming TGNB care topics, GCs were familiar with terminology and TGNB population disparities. Most participants reported having seen at least one patient within the past year they knew was TGNB (75%), and it was not uncommon for patients to disclose they were TGNB in the genetic counseling session (55%). While more than half of the GCs had personal connections to the community and felt somewhat comfortable working with TGNB patients, personal exposure to the TGNB community does not directly translate to competence and confidence in gender‐affirming care. GCs may feel exposure alone provides the tools for gender‐affirming genetic counseling; however, our data does not support this perspective. We found that personal exposure to the TGNB community was not a significant predictor of pre‐education knowledge or GCSE working with TGNB patients. This exposure could cause a GC to think they would not need additional education, like *Amplify*. Similarly, clinical exposure to a single patient who identifies as TGNB does not in itself give a counselor fluency and self‐efficacy to provide gender‐affirming, inclusive care.

Patient‐centered counseling is central to genetic counseling practice; however, it is not optimal when the patient has to take on the role of educator to achieve this. A majority of TGNB individuals in the United States report having to educate their health care providers on their appropriate care needs, which has been shown to lead to avoidance of the health care system (Grant et al., 2011). Education around gender‐affirming TGNB care is critical for GCs to bridge historically stigmatizing conversations around the care of the TGNB patient population. GCs desire more educational resources on gender‐affirming TGNB care. A recent study that showed 81.5% of GCs have sought education on these topics (Sheehan et al., 2020) is consistent with our finding that at least 45% of participants did not receive education in graduate training on gender‐affirming TGNB care. Of those who did receive training, only 20% had members of the TGNB community involved in development or administration of said education. It is important to identify the barriers limiting a consistent involvement of TGNB community members in GC education.

To ensure the needs of patients in the community are being represented in these interventions, it has also been shown to be important to engage TGNB community members in education development committees (Alpert et al., 2017; Holthouser et al., 2017). TGNB video testimonials were the most valuable aspect of the developed online modules when reviewing participant feedback on the overall *Amplify* experience. This suggests hearing personal accounts from the TGNB community provides a greater impact on GC learning than information alone. A prior study of over 400 GCs showed that desired educational resources on TGNB health care included online learning modules, followed by lectures and workshops (Sheehan et al., 2020). Our results demonstrate that online modules are an effective modality of gender‐affirming TGNB care education allowing TGNB voices and stories to be more accessible to GCs who may otherwise have limited exposure to members of the TGNB community.

GCs often feel unprepared for sessions with TGNB patients and report being nervous about their word choices (Zayhowski et al., 2019). It has also been shown that GCs experience discomfort when asking about patient pronouns (Berro et al., 2019). For gender‐affirming care to reach patients, education developed for GCs needs to improve self‐efficacy working with TGNB patients, not solely knowledge. This was highlighted in the pre‐*Amplify* data (78.4%). In this study, inclusion of a dedicated Slack communication platform contributed to shifting this education away from a one‐time experience; however, this platform lacked the ability for GCs to interact in real‐time with TGNB community members and apply gender‐affirming care practices. A subsequent event could be held for GCs who completed *Amplify* that were interested in additional clinical skill practice to apply what they learned in a safe environment with TGNB community members. This additive experience would continue to support self‐efficacy and allow GCs the opportunity to engage with more diverse TGNB community members.

### 4.1 Study limitations

While we were able to assess changes in knowledge and GCSE pre‐ and post‐*Amplify*, we were not able to determine if the improvements had a direct impact on clinical practice or patient outcomes from GCs who completed the training. Additionally, only 40 of the initial 82 participants who volunteered completed the post‐*Amplify* assessment. In the future, analyzing changes with a greater number of participants, at more than one time point, will allow for complex statistical analysis to further define the impact of *Amplify* on genetic counseling practice. Further, the analysis of participant comfort could have been expanded to improve clarity for participants on what was being assessed. As participation was voluntary, there may have been a priming bias of participants having significant interest in this topic, and therefore, a higher‐than‐average pre‐*Amplify* education. The percentage of study participants who reported being heterosexual (82.5%) is lower than the 2021 Professional Status Survey (92%) (National Society of Genetic Counselors (NSGC), 2021). Variability is also likely present in the degree of participant personal and professional exposure measured in the quantitative analysis. Gender is not always disclosed by patients in the health care setting. The assessment tool developed to analyze education effectiveness was novel and needs to be studied further to establish validity. The impact of implementing these modules during the COVID‐19 pandemic, if any, is unclear.

### 4.2 Practice implications

The findings of this study demonstrate the value of an online educational intervention in promoting gender‐affirming TGNB care education in the genetic counseling profession and helping GCs identify ways to improve the care they provide. For stakeholders invested in educating genetic counseling students and developing continuing education opportunities for GCs around gender‐affirming TGNB care, this study will aid in the decision‐making around education delivery, content development, and ways to engage TGNB community members. Additionally, establishing a trusting relationship between the genetic counseling and TGNB community will be vital for the field to remain cognizant of changing community needs and ensure TGNB patients are seeking genetic counseling services, when necessary, for their care.

### 4.3 Research recommendations

While a majority of *Amplify* was generalizable to GCs practicing in all specialties, one module focused on the cancer risk assessment and included an extensive case example in the cancer genetic counseling setting. This intervention could be adapted to emphasize other unique specialty considerations and examples. It has recently been highlighted that preconception and prenatal genetic counseling has many specific areas that could be more gender inclusive (Ruderman et al., 2021). An adapted form of *Amplify* could compile these considerations with TGNB community member experiences and examples to further educate prenatal GCs on gender‐affirming TGNB care. Additionally, data from TGNB patients surrounding patient satisfaction with GCs who have completed *Amplify* would be valuable to define the impact of the education on patient care.

## 5 CONCLUSIONS

This study was successful at developing, implementing, and analyzing an approach to gender‐affirming TGNB care education for GCs and found that online learning modules are effective at improving knowledge and GCSE. Analyzing knowledge attainment and self‐efficacy provided additional insight into how the developed education may transfer into future gender‐affirming care provided by GCs to TGNB patients. Gender‐affirming TGNB care education is lacking for GCs and the TGNB community is not consistently being included in these educational endeavors. GCs claim to be comfortable working with TGNB patients but have skills and knowledge in need of continued growth. *Amplify* is impactful for practicing GCs of all specialties to improve the care they provide to patients and can inform future decision‐making about the development of gender‐affirming care education for GCs.

## AUTHOR CONTRIBUTIONS

N.H., B.B.H., D.R.K., and B.M.Y. designed the study. N.H. executed participant recruitment and data collection. N.H. performed statistical analysis. N.H., B.B.H., D.R.K., and B.M.Y. analyzed the data. N.H. wrote the manuscript with critical feedback from all authors. B.Y. supervised the overall project. Authors N.H., B.B.H., D.R.K., and B.M.Y. confirm that they had full access to all the data in the study and take responsibility for the integrity of the data and the accuracy of the data analysis. All of the authors gave final approval of this version to be published and agree to be accountable for all aspects of the work in ensuring that questions related to the accuracy or integrity of any part of the work are appropriately investigated and resolved.

## COMPLIANCE WITH ETHICAL STANDARDS

### Conflict of interest statement

Nicole Huser, Diane R. Koeller, and Beverly M. Yashar declare that they have no conflict of interest. Bailey B. Hulswit is an employee of InheRET, Inc.

### Human studies and informed consent

This study was reviewed and granted an exemption by the University of Michigan Institutional Review Board. All procedures followed were in accordance with the ethical standards of the responsible committee on human experimentation (institutional and national) and with the Helsinki Declaration of 1975, as revised in 2000. Informed consent was obtained from individuals who participated in this study.

### Animal studies

No non‐human animal studies were carried out by the authors for this article.

### Data availability statement

The data that support the findings of this study are available from the corresponding author upon reasonable request.

## Supporting information

Supplementary Material
