# Supplementary material for: Improving gender‐affirming care in genetic counseling: Using educational tools that amplify transgender and/or gender non‐binary community voices
Source: J Genet Couns. 2022 Apr 23;31(5):1102–12. doi: 10.1002/jgc4.1581 (PMC9790640; doi:10.1002/jgc4.1581)
Supplement: Supplementary file 1 — Supplementary Material [file JGC4-31-1102-s001.docx]

| Supplemental Table 1 Genetic counseling self-efficacy assessment | | |
| --- | --- | --- |
| Competency category | Number of assessed competencies | Competency category description |
| Information Gathering | 3 | Collecting a family and medical history in a genetic counseling session from a patient who is TGNB. |
| Genetic Testing | 7 | Facilitating, interpreting, and incorporating genetic testing information into a genetic counseling session with a patient who is TGNB. |
| Case Management | 6 | Skills in case management and follow-up with patients who is TGNB. |
| Genetic Counseling Process | 5 | Implementing and explaining the process of genetic counseling during a session with a patient who is TGNB. |
| Psychosocial Counseling | 7 | Basic psychosocial assessment and counseling skills within a genetic counseling session with a patient who is TGNB. |
| Communication | 7 | Professional and interpersonal skills related to communication with a patient who is TGNB. |
| ^Categories adapted based on the validated Genetic Counseling Self-Efficacy Scale (Caldwell et al., 2018; Keller et al., 2019). TGNB: transgender and/or non-binary.^ | | |

Screenshots of *Amplify* Learning Modules


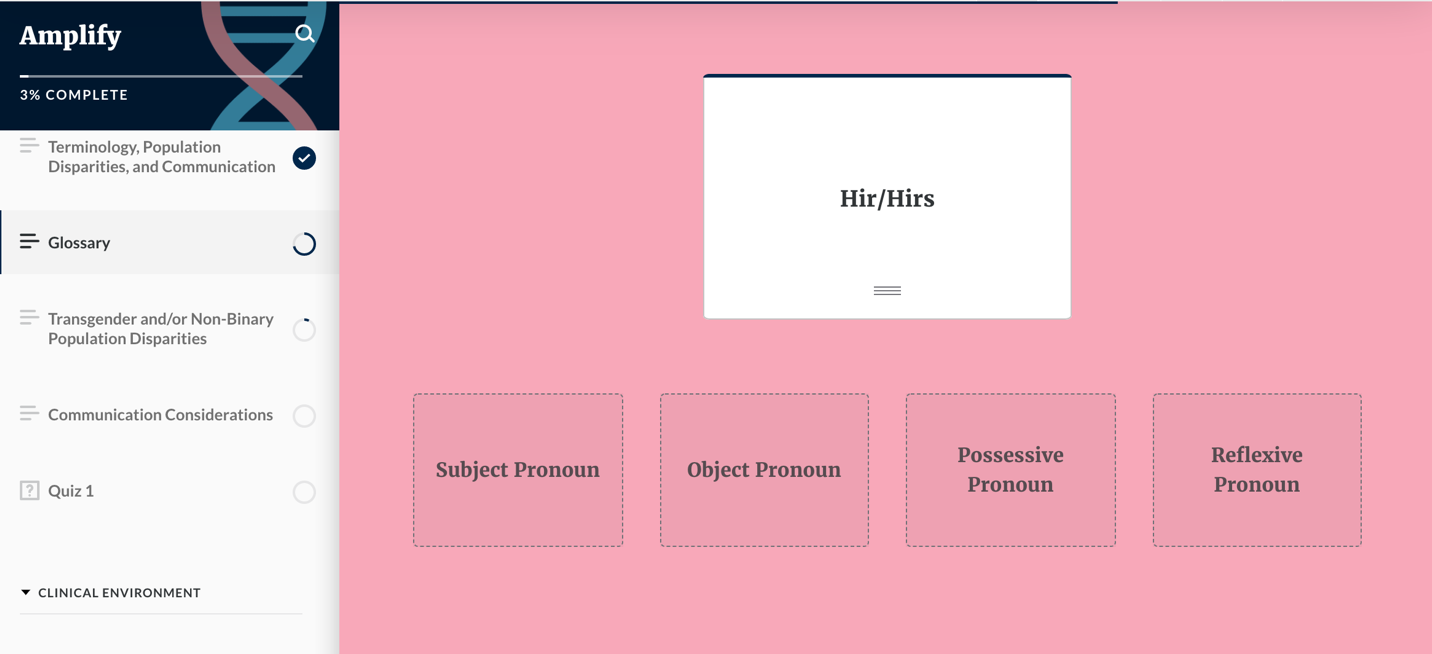


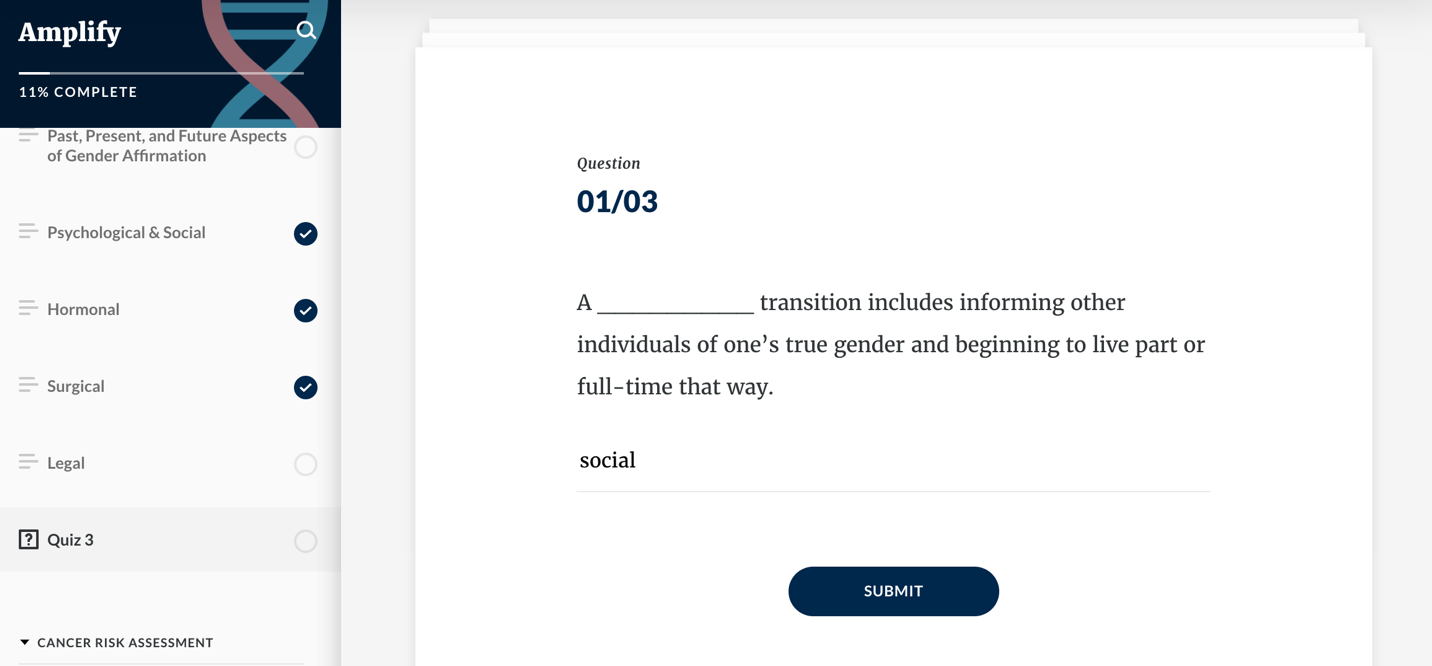


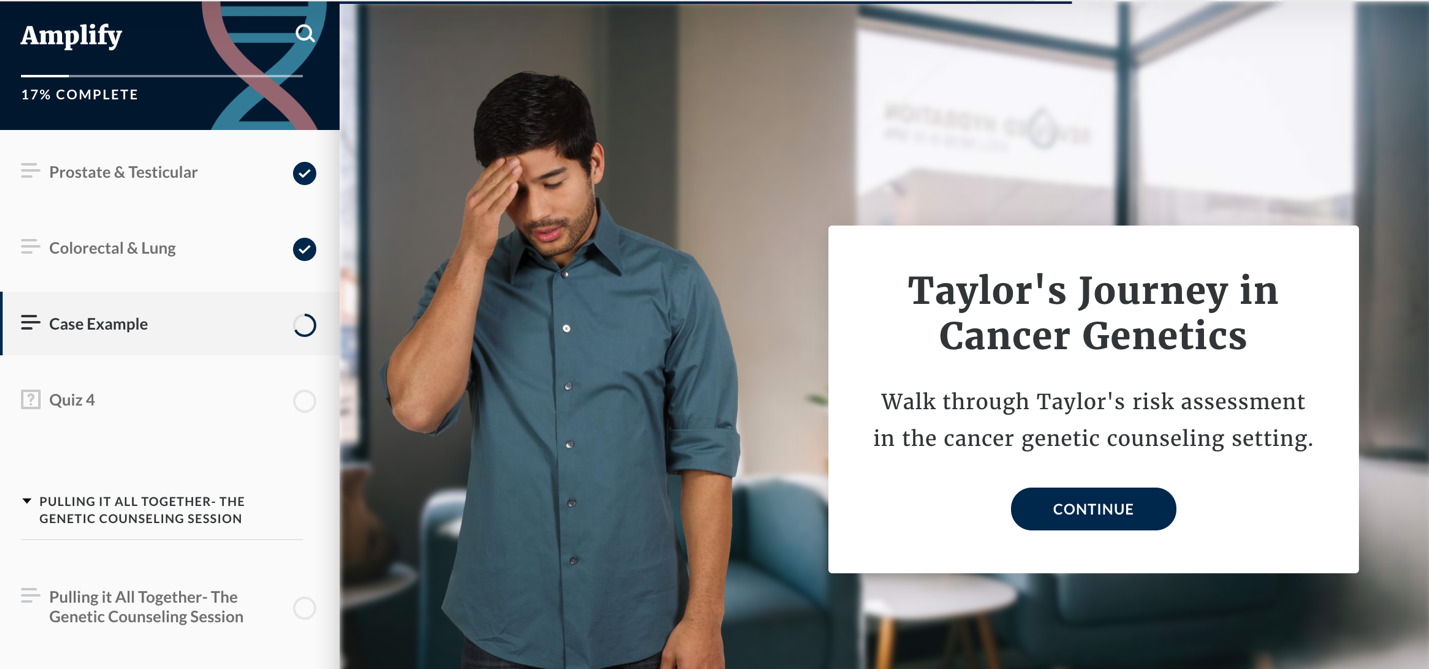


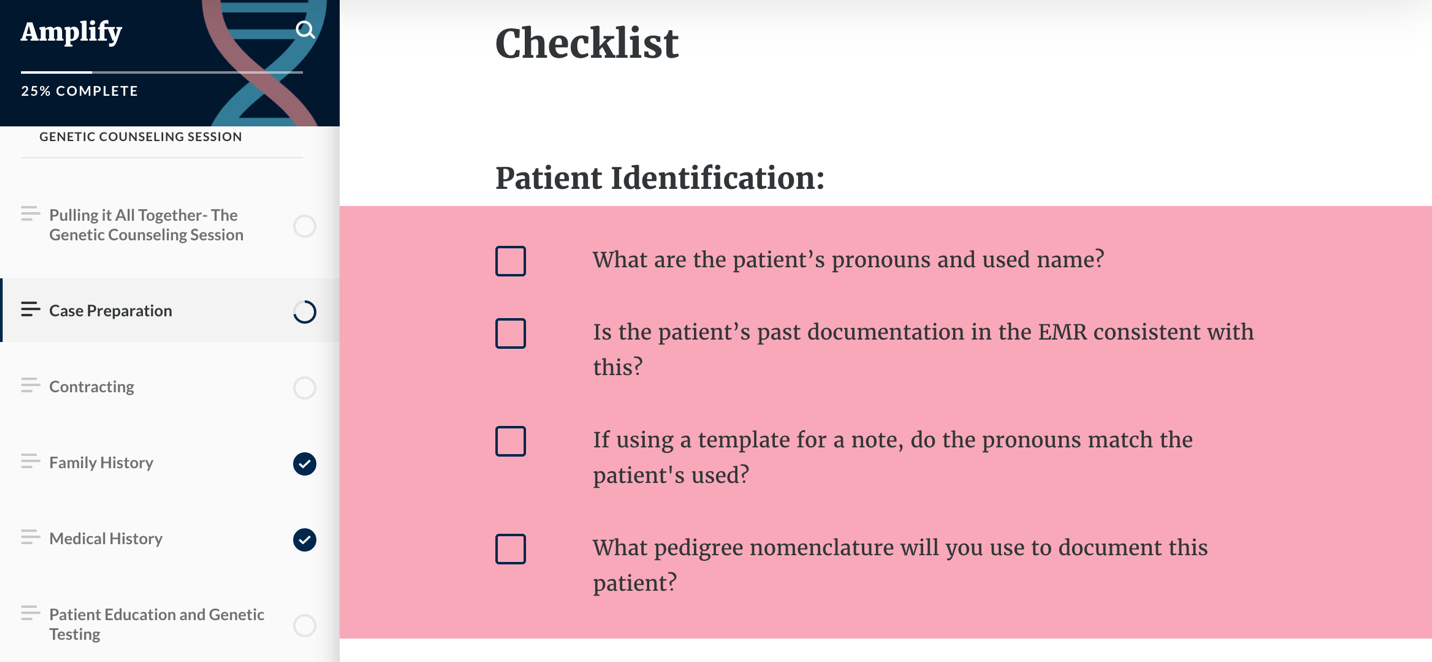


Demographic and Screening Survey

**Amplifying Transgender and Gender Non-Binary Voices in Genetic Counseling Gender-Affirming Care Education** **HUM00186581**

Thank you for your interest in this research study being conducted at the University of Michigan. The purpose of this study is to pilot a multicomponent, tailored educational program for genetic counselors around transgender and/or gender non-binary care. This will inform subsequent decision-making about the most effective methodologies to adopt in a tailored educational program.

The study consists of four parts- an online survey, a pre-education knowledge assessment, completion of an online education program, and a post-education assessment. The survey will help us determine who is eligible to participate in the on-line education program. It will evaluate your time in practice and exposure to the transgender and/or gender non-binary population. This survey should take approximately 5 minutes. The estimated time commitment for all parts of this study is 1 hour and 30 minutes. You do not need to complete all this work in one sitting.

Participating in this study is completely voluntary.  Even if you decide to participate now, you may change your mind and stop at any time.  You may choose not to answer any survey question for any reason.

**Benefits of Participating:** If you complete this survey and are selected to participate in the online education program you will obtain a free, tailored education that walks through the following units:

**Unit 1: Transgender and/or Gender Non-Binary Population Overview and Communication** **Unit 2: Gender-Affirming Clinical Environments**

**Unit 3: Past, Present, and Future Aspects of Gender Affirmation**

**Unit 4: Cancer Risk Assessment**

**Unit 5: Pulling It All Together- The Cancer Genetic Counseling Session**

Participants who complete all parts of the study will be eligible to enter a lottery to receive one of ten $25 gift cards. Participants will also be given access to all the educational content and an extensive list of helpful resources following completion. Learners will have the opportunity to join an online communication platform (Slack) community to engage with other participants during and after the completion of the online education program. Our goal is to create a community of gender-affirming care practices in genetic counseling.

**Risk of Participating:** Your survey responses will be de-identified to determine eligibility, along with subsequent data if you are chosen to participate in the online learning program. A small risk exists that your responses will be linked with personally identifiable information, unintentionally. We will take every precaution to uphold confidentiality. The de-identified information will only be used for the purposes of this research study. Identifiable information will only be used to coordinate further participation in the multiple study components. All information will be stored on secure servers at the University of Michigan.

**If you have any questions or concerns, please contact the principal investigator, Nicole Huser (nmhuser@umich.edu).**

Q1 Do you consent to be a part of this study?

- I consent to participate in this research study.
- I do not consent to participate in this research study.

Skip To: End of Survey If Do you consent to be a part of this study? = I do not consent to participate in this research study.

Q2 Which of the following best describes you?

- Certified Genetic Counselor
- Board-Eligible Genetic Counselor
- Genetic Counseling Graduate Student

Q3 How many years of experience do you have practicing genetic counseling in total?

________________________________________________________________

Q4 What specialty do you practice in?

- Cancer
- Prenatal
- Pediatrics
- General Genetics
- Preconception
- Other ________________________________________________

Q5 How many years total have you practiced in this specialty?

________________________________________________________________

Q6 Approximately how many patients do you see per month?

- Fewer than 5 patients
- 5-15 patients
- 16-30 patients
- More than 30 patients

Q7 Approximately how many patients have you seen in practice, in total, that you were aware identified as transgender and/or non-binary?

**Transgender (adjective):** Describes a person whose gender identity and sex assigned at birth do not correspond based on traditional expectations
**Non-Binary (adjective):** Describes a person whose gender identity falls outside of the traditional gender binary structure of girl/woman and boy/man

________________________________________________________________

Q8 Has a patient ever disclosed to you they identify as transgender and/or gender non-binary during a genetic counseling session?

- Yes
- Maybe
- No

Q9 Do you work in a clinic specifically serving patients from the transgender and/or gender non-binary community?

- Yes
- Maybe
- No

| Page Break |  |
| --- | --- |

Q10 Do you personally know someone who identifies as transgender and/or gender non-binary?

- Yes
- Maybe
- No

Q11 Did you receive education on transgender and/or gender non-binary care considerations during graduate school?

- Yes
- Maybe
- No

Display This Question:

If Did you receive education on transgender and/or gender non-binary care considerations during grad... = Yes

Q12 What did that education consist of? (select all that apply)

- Single lecture
- Integrated throughout courses within program
- Online learning module
- Workshop
- No specific education
- Other ________________________________________________

Display This Question:

If Did you receive education on transgender and/or gender non-binary care considerations during grad... = Yes

Q13 Were members of the transgender and/or gender non-binary community involved in developing and/or administering that education?

- Yes
- Maybe
- No

Q14 What education regarding transgender and/or gender non-binary care considerations have you received following graduate training? (select all that apply)

- Lecture
- Workshop
- Informed by colleagues
- Internet sources
- Articles or books
- No specific education
- Other ________________________________________________

Q15 How comfortable do you feel working with transgender and/or non-binary patients?

- Extremely comfortable
- Somewhat comfortable
- Neither comfortable nor uncomfortable
- Somewhat uncomfortable
- Extremely uncomfortable

Q16 How comfortable do you feel assessing the cancer risk of an individual who identifies as transgender and/or non-binary?

- Extremely comfortable
- Somewhat comfortable
- Neither comfortable nor uncomfortable
- Somewhat uncomfortable
- Extremely uncomfortable

| Page Break |  |
| --- | --- |

Q17 What is your age?

- Under 18
- 18 - 24
- 25 - 34
- 35 - 44
- 45 - 54
- 55 - 64
- 65 - 74
- 75 - 84
- 85 or older

Q18 What is your gender identity?

- Cisgender man
- Cisgender woman
- Transgender man
- Transgender woman
- Non-Binary
- Prefer to self-describe ________________________________________________
- Prefer not to say

Q19 What is your sexual orientation?

- Heterosexual
- Homosexual
- Bisexual
- Queer
- Prefer to self-describe ________________________________________________
- Prefer not to say

Q20 What is your race?

- American Indian or Alaska Native
- Asian
- Asian Indian
- Black or African American
- Middle Eastern or North African
- Native Hawaiian or Pacific Islander
- White
- Other ________________________________________________
- Prefer not to say

Q21 What is your current NSGC region of practice?

- Region 1: CT, MA, ME, NH, RI, VT, CN Maritime Provinces
- Region 2: DC, DE, MD, NJ, NY, PA, VA, WV, PR, VI, Quebec
- Region 3: AL, FL, GA, KY, LA, MS, NC, SC, TN
- Region 4: AR, IA, IL, IN, KS, MI, MN, MO, ND, NE, OH, OK, SD, WI, Ontario
- Region 5: AZ, CO, MT, NM, TX, UT, WY, Alberta, Manitoba, Saskatchewan
- Region 6: AK, CA, HI, ID, NV, OR, WA, British Columbia

Q22 What genetic counseling graduate program do/did you attend?

- Name of program: ________________________________________________
- Prefer not to say

Q23 What year did/will you graduate from a genetic counseling graduate program?

- Year ________________________________________________
- Prefer not to say

Q24 Are you willing to participate in a 60-90-minute online educational program about gender-affirming care in genetic counseling and transgender and/or gender non-binary care considerations?

- Yes
- No

Display This Question:

If Are you willing to participate in a 60-90-minute online educational program about gender-affirmin... = Yes

Q25 Please provide an email for us to add you to the Canvas course page of the educational program as well as an online virtual community (Slack) to utilize during and after the educational program. This virtual community will serve as a safe space for conversations and to brainstorm ways to make genetic counseling more gender-inclusive.

After providing this email, you will be redirected to the pre-education assessment survey, estimated to take 10-15 minutes. After this assessment, look in your email (possibly Spam folder), for the invitation to create an account to do the program on your own time. This email should arrive within the next 24 hours.

This email will be dissociated from your survey responses. As a reminder, you can go back into this survey and choose not to answer any survey question for any reason.

___________________________________________________________________________

Pre/Post Module Assessment

**Amplifying Transgender and Gender Non-Binary Voices in Genetic Counseling Gender-Affirming Care Education** **HUM00186581**

Thank you for completing *Amplify* - an educational program tailored to genetic counselors on gender-affirming care and TGNB care considerations.

The following is a pre(or post)-education quantitative assessment to assess any changes after completing the online learning module. This will ask questions to assess your self-efficacy counseling TGNB individuals and objective knowledge. This should take approximately 15 minutes.

Participating in this study is completely voluntary.  Even if you decide to participate now, you may change your mind and stop at any time.

Benefits of Participating:

Participants who complete this survey will be eligible to enter a lottery to possibly receive one of 10 $25 gift cards. Participants will also be given access to all information provided and compiled resources in a PDF format, following completion. You will also continue to have the opportunity to join and engage in the Slack group with other participants on topics surrounding gender-affirming care practices in genetic counseling.

Risk of Participating: Your responses will be de-identified. A small risk exists that your responses will be linked with personally identifiable information, unintentionally. We will take every precaution to uphold confidentiality. The de-identified information will only be used for the purposes of this research study. All information will be stored on secure servers at the University of Michigan.

**If you have any questions or concerns, please contact the principal investigator, Nicole Huser (nmhuser@med.umich.edu).**

**The following questions are assessing the baseline knowledge you have around gender-affirming care in the counseling setting and consideration for transgender and/or non-binary patients. If you are unfamiliar with a concept and therefore, do not know how to approach answering a question, please select "I am unfamiliar with this concept." All content addressed in these questions will be covered in the educational modules to follow.**

Q1   __________ is a person’s inner sense of being a woman, man, other, or having no gender. _________ is the way a person communicates their gender to the world (ex: clothing, speech, behavior).

- gender expression ; gender identity
- gender fluid ; gender expression
- gender identity ; gender expression
- gender identity ; gender fluid
- I am unfamiliar with this concept

Q2 What is the preferred term to replace biological female/male? This notation may be integrated into pedigree nomenclature and documentation.

- Assigned female/male at birth (AFAB/AMAB)
- Biological female/male is the appropriate nomenclature
- Female/male
- XX/XY
- I am unfamiliar with this concept

Q3 For an individual to identify as transgender and/or non-binary, they must undergo some form of gender-affirming hormone therapy and/or gender-affirming surgery.

- True
- False
- I am unfamiliar with this concept

Q4 What are the steps to take when you misgender someone?

- Acknowledge the mistake, apologize sincerely, and commit to improving next time
- Apologize and move on in the conversation
- Apologize to the individual and explain what may have led to the mistake
- Ignore the mistake but internally commit to improving next time
- I am unfamiliar with this concept

Q5 When planning to ask a patient about their pronouns are, what is an important consideration?

- Does their gender expression make it obvious what their pronouns are?
- Have the patient’s pronouns already been documented, such as on a patient in-take form?
- Is their gender expression unique?
- Will they understand what I am asking?
- I am unfamiliar with this concept

| Page Break |  |
| --- | --- |

Q6 If there are discrepancies in a patient’s past notes in the EMR regarding their name used or pronouns, which of the following would be appropriate action to take?

- Advocate for corrections and consistency from the other providers the patient was seen by
- Focus on the accuracy of your own documentation, other providers will correct their mistakes
- Keep the documentation consistent with the name and pronouns past providers have used
- Wait until legal documents reflect a patient’s chosen name and gender identity before making any changes
- I am unfamiliar with this concept

Q7 What aspects of care in a cancer genetic counseling clinic would likely need to be examined more closely for gender inclusivity?

- Genetic testing options
- Presentation of cancer risks
- Waiting areas available for a patient to use in clinic
- I am unfamiliar with this concept

Q8 Transgender and/or non-binary patients could identify that you are a gender-inclusive provider before being seen by all of the following except:

- Genetic counseling clinic website or webpage
- Organization mission statement
- Provider credentials
- Referring providers
- I am unfamiliar with this concept

Q9 The bathroom of a clinic can show a patient how inclusive clinic providers are.

- True
- False
- I am unfamiliar with this concept

Q10 Art, magazines, and pamphlets in a waiting area can show a patient how inclusive the providers at a clinic are.

- True
- False
- I am unfamiliar with this concept

| Page Break |  |
| --- | --- |

Q11 What is the most up-to-date term used by the Diagnostic and Statistical Manual of Mental Disorders (DSM-5) to describe when an individual’s physical and assigned gender is different from the one they identify with?

- Gender complex
- Gender dysphoria
- Gender fluid
- Gender identity disorder
- I am unfamiliar with this concept

Q12 When an individual informs others of their true gender and begins to live partially or full-time that way, what type of transition is occurring?

- Emotional transition
- Physical transition
- Psychological transition
- Social transition
- I am unfamiliar with this concept

Q13 The range of reproductive desires of transgender and/or non-binary individuals are the same as cisgender individuals.

- True
- False
- I am unfamiliar with this concept

Q14 Which of the following is a masculinizing surgical option?

- Breast augmentation
- Hysterectomy
- Orchiectomy
- Thyroid cartilage reduction
- I am unfamiliar with this concept

Q15 When a legal document reflects one's gender identity, this contributes to an individual’s health in all of the following ways except:

- Access to medical care
- Safety during travel
- Safety in their living environment
- Supporting employment
- I am unfamiliar with this concept

| Page Break |  |
| --- | --- |

Q16 Of the organs listed below, all of the following would be important to know are present in a patient when assessing their cancer risks except:

- Breast/chest tissue
- Ovaries
- Penis
- Prostate
- I am unfamiliar with this concept

Q17 A transgender woman on progesterone is suspected to have an _______ risk for breast cancer. This risk is ________ when taken with estrogen.

- Decreased ; decreased
- Decreased ; increased
- Increased ; decreased
- Increased ; increased
- I am unfamiliar with this concept

Q18 A __________ removes and/or constructs a person’s chest to be more aligned with that person’s gender identity, while a ___________ removes chest tissue to reduce the risk of developing breast cancer or breast cancer recurrence.

- Gender-affirming chest surgery ; risk-reducing bilateral mastectomy
- Risk-reducing bilateral mastectomy ; gender-affirming chest surgery
- Risk-reducing bilateral mastectomy ; top surgery
- Top surgery ; Bottom surgery
- I am unfamiliar with this concept

Q19 A transgender woman on estrogen and progesterone has an increased risk for ovarian cancer.

- True
- False
- I am unfamiliar with this concept

Q20 Your patient, Jessie is a transgender man who was recently diagnosed with Lynch syndrome after an MSH2 pathogenic variant was identified on genetic testing. 
**Which of the following would be relevant and appropriate information to elicit from Jessie?**

- Colonoscopy screening
- Hormone therapy to increase estrogen levels
- Prostate cancer screening
- I am unfamiliar with this concept

| Page Break |  |
| --- | --- |

Q21 When preparing for a patient who you know identifies as transgender and/or gender non-binary, which of the following questions would be important to answer in case preparation that would not be done if you knew your patient was cisgender?

- Are there gender-affirming clinics or providers you have to recommend to the patient?
- Has the patient undergone any surgeries that impact their cancer risk?
- If using a template for a note, do the pronouns match the patient’s?
- What organs are present in the patient?
- I am unfamiliar with this concept

Q22 A patient indicates use of ze/hir/hirs pronouns. 
**How should the following sentence be written in your note about the encounter?**“______  was diagnosed with prostate cancer at 57. ______ father had colon cancer at the age of 71.”

- Hir ; hirs
- Hir ; ze
- Ze ; hir
- Ze ; hirs
- I am unfamiliar with this concept

Q23 If an individual on a pedigree was documented with a square and the note AFAB, what gender identity is most likely closest to the one they identify with?

- Cisgender man
- Non-Binary
- Transgender man
- Transgender woman
- I am unfamiliar with this concept

Q24 Your patient identifies as a transgender woman. 
**Which of the follow would be the most informative and affirming way to document your patient on the pedigree for another provider to interpret?**

- Circle
- Circle with AMAB
- Diamond
- Square
- Square within a circle
- I am unfamiliar with this concept

Q25 A visual aid is likely to be gender-inclusive with content covering all of the following subjects except:

- Cancer risks for an individual with FAP
- Causes of cancer
- DNA -> Gene -> Proteins
- Inheritance
- I am unfamiliar with this concept

End of Block: Default Question Block

Start of Block: GCSE

**A number of competencies are described below that may be part of a genetic counseling session. Please rate how certain you are that you can independently perform this competency today in a genetic counseling session with a patient who identifies as transgender and/or non-binary (TGNB).**

Transgender (adjective): Describes a person whose gender identity and sex assigned at birth do not correspond based on traditional expectations
Non-Binary (adjective): Describes a person whose gender identity falls outside of the traditional gender binary structure of girl/woman and boy/man

Q26 Accurately record a family history by drawing a pedigree using appropriate pedigree symbols with a client who identifies as TGNB.

|  | **Not at all certain** | **Moderately certain** | **Highly certain** |
| --- | --- | --- | --- |

|  | 0 | 10 | 20 | 30 | 40 | 50 | 60 | 70 | 80 | 90 | 100 |
| --- | --- | --- | --- | --- | --- | --- | --- | --- | --- | --- | --- |

| Independently Perform Competency | 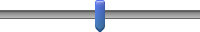 |
| --- | --- |

Q27 Ask targeted, structured questions pertinent to an individual case in order to elicit a family history with a client who identifies as TGNB.

|  | **Not at all certain** | **Moderately certain** | **Highly certain** |
| --- | --- | --- | --- |

|  | 0 | 10 | 20 | 30 | 40 | 50 | 60 | 70 | 80 | 90 | 100 |
| --- | --- | --- | --- | --- | --- | --- | --- | --- | --- | --- | --- |

| Independently Perform Competency | 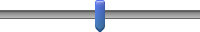 |
| --- | --- |

Q28 Ask targeted questions in order to elicit pertinent medical history from a client who identifies as TGNB.

|  | **Not at all certain** | **Moderately certain** | **Highly certain** |
| --- | --- | --- | --- |

|  | 0 | 10 | 20 | 30 | 40 | 50 | 60 | 70 | 80 | 90 | 100 |
| --- | --- | --- | --- | --- | --- | --- | --- | --- | --- | --- | --- |

| Independently Perform Competency | 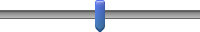 |
| --- | --- |

Q29 Assess the accuracy with which a diagnostic genetic/genomic test identifies the clinical status (clinical validity) of a client who identifies as TGNB.

|  | **Not at all certain** | **Moderately certain** | **Highly certain** |
| --- | --- | --- | --- |

|  | 0 | 10 | 20 | 30 | 40 | 50 | 60 | 70 | 80 | 90 | 100 |
| --- | --- | --- | --- | --- | --- | --- | --- | --- | --- | --- | --- |

| Independently Perform Competency | 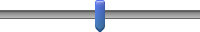 |
| --- | --- |

Q30 Assess the value of a diagnostic genetic/genomic test for determining treatment and management (clinical utility) for a client who identifies as TGNB.

|  | **Not at all certain** | **Moderately certain** | **Highly certain** |
| --- | --- | --- | --- |

|  | 0 | 10 | 20 | 30 | 40 | 50 | 60 | 70 | 80 | 90 | 100 |
| --- | --- | --- | --- | --- | --- | --- | --- | --- | --- | --- | --- |

| Independently Perform Competency | 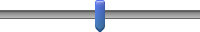 |
| --- | --- |

| Page Break |  |
| --- | --- |

Q31 Select the most appropriate laboratory and genetic/genomic test for the given clinical situation with a client who identifies as TGNB.

|  | **Not at all certain** | **Moderately certain** | **Highly certain** |
| --- | --- | --- | --- |

|  | 0 | 10 | 20 | 30 | 40 | 50 | 60 | 70 | 80 | 90 | 100 |
| --- | --- | --- | --- | --- | --- | --- | --- | --- | --- | --- | --- |

| Independently Perform Competency | 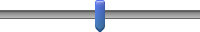 |
| --- | --- |

Q32 Discuss potential benefits, risks, and limitations of genetic/genomic testing with a client who identifies as TGNB.

|  | **Not at all certain** | **Moderately certain** | **Highly certain** |
| --- | --- | --- | --- |

|  | 0 | 10 | 20 | 30 | 40 | 50 | 60 | 70 | 80 | 90 | 100 |
| --- | --- | --- | --- | --- | --- | --- | --- | --- | --- | --- | --- |

| Independently Perform Competency | 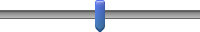 |
| --- | --- |

Q33 Facilitate the ordering of appropriate genetic/genomic testing for a client who identifies as TGNB.

|  | **Not at all certain** | **Moderately certain** | **Highly certain** |
| --- | --- | --- | --- |

|  | 0 | 10 | 20 | 30 | 40 | 50 | 60 | 70 | 80 | 90 | 100 |
| --- | --- | --- | --- | --- | --- | --- | --- | --- | --- | --- | --- |

| Independently Perform Competency | 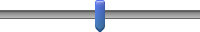 |
| --- | --- |

Q34 Interpret the clinical implications of genetic/genomic test reports for a client who identifies as TGNB.

|  | **Not at all certain** | **Moderately certain** | **Highly certain** |
| --- | --- | --- | --- |

|  | 0 | 10 | 20 | 30 | 40 | 50 | 60 | 70 | 80 | 90 | 100 |
| --- | --- | --- | --- | --- | --- | --- | --- | --- | --- | --- | --- |

| Independently Perform Competency | 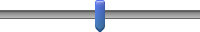 |
| --- | --- |

Q35 Evaluate familial implications from genetic/genomic test results for a client who identifies as TGNB.

|  | **Not at all certain** | **Moderately certain** | **Highly certain** |
| --- | --- | --- | --- |

|  | 0 | 10 | 20 | 30 | 40 | 50 | 60 | 70 | 80 | 90 | 100 |
| --- | --- | --- | --- | --- | --- | --- | --- | --- | --- | --- | --- |

| Independently Perform Competency | 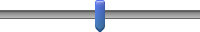 |
| --- | --- |

| Page Break |  |
| --- | --- |

Q36 Incorporate the results of screening, testing, family history, environmental and lifestyle factors, and other relevant information to provide an accurate risk assessment for clients who identify as TGNB (e.g. probability of carrier status, cancer risk assessment).

|  | **Not at all certain** | **Moderately certain** | **Highly certain** |
| --- | --- | --- | --- |

|  | 0 | 10 | 20 | 30 | 40 | 50 | 60 | 70 | 80 | 90 | 100 |
| --- | --- | --- | --- | --- | --- | --- | --- | --- | --- | --- | --- |

| Independently Perform Competency | 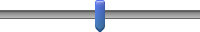 |
| --- | --- |

Q37 Modify the case management plan as needed for a client who identifies as TGNB in order to incorporate changes in management and surveillance recommendations.

|  | **Not at all certain** | **Moderately certain** | **Highly certain** |
| --- | --- | --- | --- |

|  | 0 | 10 | 20 | 30 | 40 | 50 | 60 | 70 | 80 | 90 | 100 |
| --- | --- | --- | --- | --- | --- | --- | --- | --- | --- | --- | --- |

| Independently Perform Competency | 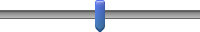 |
| --- | --- |

Q38 Present the genetic counseling encounter with a client who identifies as TGNB orally to other health care providers in a clear and concise manner (e.g. case presentation to physician).

|  | **Not at all certain** | **Moderately certain** | **Highly certain** |
| --- | --- | --- | --- |

|  | 0 | 10 | 20 | 30 | 40 | 50 | 60 | 70 | 80 | 90 | 100 |
| --- | --- | --- | --- | --- | --- | --- | --- | --- | --- | --- | --- |

| Independently Perform Competency | 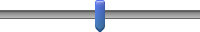 |
| --- | --- |

Q39 Document the genetic counseling encounter with a client who identifies as TGNB in accordance with professional guidelines and standards (e.g. medical record documentation; letters to other providers).

|  | **Not at all certain** | **Moderately certain** | **Highly certain** |
| --- | --- | --- | --- |

|  | 0 | 10 | 20 | 30 | 40 | 50 | 60 | 70 | 80 | 90 | 100 |
| --- | --- | --- | --- | --- | --- | --- | --- | --- | --- | --- | --- |

| Independently Perform Competency | 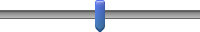 |
| --- | --- |

Q40 Identify appropriate resources, services and support for a client who identifies as TGNB.

|  | **Not at all certain** | **Moderately certain** | **Highly certain** |
| --- | --- | --- | --- |

|  | 0 | 10 | 20 | 30 | 40 | 50 | 60 | 70 | 80 | 90 | 100 |
| --- | --- | --- | --- | --- | --- | --- | --- | --- | --- | --- | --- |

| Independently Perform Competency | 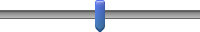 |
| --- | --- |

| Page Break |  |
| --- | --- |

Q41 Establish a mutually agreed upon genetic counseling agenda with a client who identifies as TGNB.

|  | **Not at all certain** | **Moderately certain** | **Highly certain** |
| --- | --- | --- | --- |

|  | 0 | 10 | 20 | 30 | 40 | 50 | 60 | 70 | 80 | 90 | 100 |
| --- | --- | --- | --- | --- | --- | --- | --- | --- | --- | --- | --- |

| Independently Perform Competency | 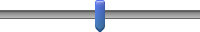 |
| --- | --- |

Q42 Explain the genetic counseling process to a client who identifies as TGNB.

|  | **Not at all certain** | **Moderately certain** | **Highly certain** |
| --- | --- | --- | --- |

|  | 0 | 10 | 20 | 30 | 40 | 50 | 60 | 70 | 80 | 90 | 100 |
| --- | --- | --- | --- | --- | --- | --- | --- | --- | --- | --- | --- |

| Independently Perform Competency | 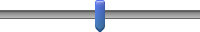 |
| --- | --- |

Q43 Contract with a client who identifies as TGNB throughout the encounter to address emerging concerns.

|  | **Not at all certain** | **Moderately certain** | **Highly certain** |
| --- | --- | --- | --- |

|  | 0 | 10 | 20 | 30 | 40 | 50 | 60 | 70 | 80 | 90 | 100 |
| --- | --- | --- | --- | --- | --- | --- | --- | --- | --- | --- | --- |

| Independently Perform Competency | 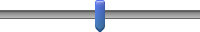 |
| --- | --- |

Q44 Assess pertinent information relating to psychosocial history such as a client who identifies as TGNB’s emotions, individual and family experiences, beliefs, behaviors, values, coping mechanisms, and adaptive capabilities.

|  | **Not at all certain** | **Moderately certain** | **Highly certain** |
| --- | --- | --- | --- |

|  | 0 | 10 | 20 | 30 | 40 | 50 | 60 | 70 | 80 | 90 | 100 |
| --- | --- | --- | --- | --- | --- | --- | --- | --- | --- | --- | --- |

| Independently Perform Competency | 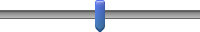 |
| --- | --- |

Q45 Respond to a client who identifies as TGNB’s emotional and behavioral cues, expressed both verbally and non-verbally.

|  | **Not at all certain** | **Moderately certain** | **Highly certain** |
| --- | --- | --- | --- |

|  | 0 | 10 | 20 | 30 | 40 | 50 | 60 | 70 | 80 | 90 | 100 |
| --- | --- | --- | --- | --- | --- | --- | --- | --- | --- | --- | --- |

| Independently Perform Competency | 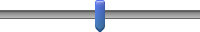 |
| --- | --- |

| Page Break |  |
| --- | --- |

Q46 Respond to a client who identifies as TGNB using normalization in a genetic counseling session.

|  | **Not at all certain** | **Moderately certain** | **Highly certain** |
| --- | --- | --- | --- |

|  | 0 | 10 | 20 | 30 | 40 | 50 | 60 | 70 | 80 | 90 | 100 |
| --- | --- | --- | --- | --- | --- | --- | --- | --- | --- | --- | --- |

| Independently Perform Competency | 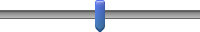 |
| --- | --- |

Q47 Utilize anticipatory guidance in a genetic counseling session with a client who identifies as TGNB.

|  | **Not at all certain** | **Moderately certain** | **Highly certain** |
| --- | --- | --- | --- |

|  | 0 | 10 | 20 | 30 | 40 | 50 | 60 | 70 | 80 | 90 | 100 |
| --- | --- | --- | --- | --- | --- | --- | --- | --- | --- | --- | --- |

| Independently Perform Competency | 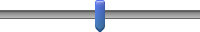 |
| --- | --- |

Q48 Utilize advanced empathy in a genetic counseling session with a client who identifies as TGNB.

|  | **Not at all certain** | **Moderately certain** | **Highly certain** |
| --- | --- | --- | --- |

|  | 0 | 10 | 20 | 30 | 40 | 50 | 60 | 70 | 80 | 90 | 100 |
| --- | --- | --- | --- | --- | --- | --- | --- | --- | --- | --- | --- |

| Independently Perform Competency | 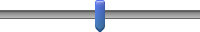 |
| --- | --- |

Q49 Utilize in-depth exploration of a client who identifies as TGNB’s responses to risks and options.

|  | **Not at all certain** | **Moderately certain** | **Highly certain** |
| --- | --- | --- | --- |

|  | 0 | 10 | 20 | 30 | 40 | 50 | 60 | 70 | 80 | 90 | 100 |
| --- | --- | --- | --- | --- | --- | --- | --- | --- | --- | --- | --- |

| Independently Perform Competency | 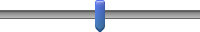 |
| --- | --- |

Q50 Evaluate the need for intervention and referral for a client who identifies as TGNB based on their psychosocial needs.

|  | **Not at all certain** | **Moderately certain** | **Highly certain** |
| --- | --- | --- | --- |

|  | 0 | 10 | 20 | 30 | 40 | 50 | 60 | 70 | 80 | 90 | 100 |
| --- | --- | --- | --- | --- | --- | --- | --- | --- | --- | --- | --- |

| Independently Perform Competency | 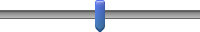 |
| --- | --- |

| Page Break |  |
| --- | --- |

Q51 Implement evidence-based models of counseling to a genetic counseling session with a client who identifies as TGNB as appropriate (e.g. short-term client-centered counseling, grief counseling, crisis counseling).

|  | **Not at all certain** | **Moderately certain** | **Highly certain** |
| --- | --- | --- | --- |

|  | 0 | 10 | 20 | 30 | 40 | 50 | 60 | 70 | 80 | 90 | 100 |
| --- | --- | --- | --- | --- | --- | --- | --- | --- | --- | --- | --- |

| Independently Perform Competency | 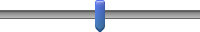 |
| --- | --- |

Q52 Facilitate client decision-making that is consistent with the values of a client who identifies as TGNB.

|  | **Not at all certain** | **Moderately certain** | **Highly certain** |
| --- | --- | --- | --- |

|  | 0 | 10 | 20 | 30 | 40 | 50 | 60 | 70 | 80 | 90 | 100 |
| --- | --- | --- | --- | --- | --- | --- | --- | --- | --- | --- | --- |

| Independently Perform Competency | 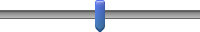 |
| --- | --- |

Q53 Maintain professional boundaries by ensuring directive statements, self-disclosure, and self-involving responses are in the best interest of the client who identifies as TGNB.

|  | **Not at all certain** | **Moderately certain** | **Highly certain** |
| --- | --- | --- | --- |

|  | 0 | 10 | 20 | 30 | 40 | 50 | 60 | 70 | 80 | 90 | 100 |
| --- | --- | --- | --- | --- | --- | --- | --- | --- | --- | --- | --- |

| Independently Perform Competency | 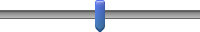 |
| --- | --- |

Q54 Respond to client-counselor relationship dynamics with a client who identifies as TGNB, such as transference and countertransference.

|  | **Not at all certain** | **Moderately certain** | **Highly certain** |
| --- | --- | --- | --- |

|  | 0 | 10 | 20 | 30 | 40 | 50 | 60 | 70 | 80 | 90 | 100 |
| --- | --- | --- | --- | --- | --- | --- | --- | --- | --- | --- | --- |

| Independently Perform Competency | 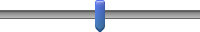 |
| --- | --- |

Q55 Respond to a client who identifies as TGNB’s cultural beliefs relevant to the genetic counseling session.

|  | **Not at all certain** | **Moderately certain** | **Highly certain** |
| --- | --- | --- | --- |

|  | 0 | 10 | 20 | 30 | 40 | 50 | 60 | 70 | 80 | 90 | 100 |
| --- | --- | --- | --- | --- | --- | --- | --- | --- | --- | --- | --- |

| Independently Perform Competency | 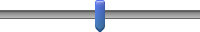 |
| --- | --- |

| Page Break |  |
| --- | --- |

Q56 Utilize risk communication principles and theory to maximize understanding for a client who identifies as TGNB’s understanding.

|  | **Not at all certain** | **Moderately certain** | **Highly certain** |
| --- | --- | --- | --- |

|  | 0 | 10 | 20 | 30 | 40 | 50 | 60 | 70 | 80 | 90 | 100 |
| --- | --- | --- | --- | --- | --- | --- | --- | --- | --- | --- | --- |

| Independently Perform Competency | 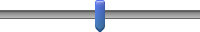 |
| --- | --- |

Q57 Communicate relevant genetic information to a client who identifies as TGNB to help them better understand certain conditions.

|  | **Not at all certain** | **Moderately certain** | **Highly certain** |
| --- | --- | --- | --- |

|  | 0 | 10 | 20 | 30 | 40 | 50 | 60 | 70 | 80 | 90 | 100 |
| --- | --- | --- | --- | --- | --- | --- | --- | --- | --- | --- | --- |

| Independently Perform Competency | 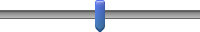 |
| --- | --- |

Q58 Communicate with clients in a way that is clear and unambiguous based on the needs and circumstances for a client who identifies as TGNB.

|  | **Not at all certain** | **Moderately certain** | **Highly certain** |
| --- | --- | --- | --- |

|  | 0 | 10 | 20 | 30 | 40 | 50 | 60 | 70 | 80 | 90 | 100 |
| --- | --- | --- | --- | --- | --- | --- | --- | --- | --- | --- | --- |

| Independently Perform Competency | 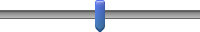 |
| --- | --- |

Q59 Present balanced descriptions of lived experiences of people with various genetic conditions to a client who identifies as TGNB.

|  | **Not at all certain** | Moderately certain | Highly certain |
| --- | --- | --- | --- |

|  | 0 | 10 | 20 | 30 | 40 | 50 | 60 | 70 | 80 | 90 | 100 |
| --- | --- | --- | --- | --- | --- | --- | --- | --- | --- | --- | --- |

| Independently Perform Competency | 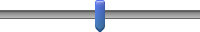 |
| --- | --- |

Q60 Respond to ethical and moral dilemmas that may arise in genetic counseling practice with a client who identifies as TGNB.

|  | Not at all certain | Moderately certain | Highly certain |
| --- | --- | --- | --- |

|  | 0 | 10 | 20 | 30 | 40 | 50 | 60 | 70 | 80 | 90 | 100 |
| --- | --- | --- | --- | --- | --- | --- | --- | --- | --- | --- | --- |

| Independently Perform Competency | 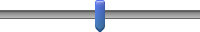 |
| --- | --- |

End of Block: GCSE

Start of Block: Block 4

Only asked on the post-module assessment:

Q61 Please provide feedback on your experience working through the online educational program Amplify:

________________________________________________________________

________________________________________________________________

________________________________________________________________

________________________________________________________________

________________________________________________________________

End of Block: Block 4
